# CDK1 is up-regulated by temozolomide in an NF-κB dependent manner in glioblastoma

**DOI:** 10.1038/s41598-021-84912-4

**Published:** 2021-03-11

**Authors:** David J. Voce, Giovanna M. Bernal, Kirk E. Cahill, Longtao Wu, Nassir Mansour, Clayton D. Crawley, Paige-Ashley S. Campbell, Ainhoa Arina, Ralph R. Weichselbaum, Bakhtiar Yamini

**Affiliations:** 1 Department of Surgery, Section of Neurosurgery, The University of Chicago, Chicago, IL 60637 USA; 2 Department of Neurosurgery, Vanderbilt University Medical Center, Nashville, TN 37232 USA; 3 Department of Radiation and Cellular Oncology, The Ludwig Center for Metastasis Research, The University of Chicago, Chicago, IL 60637 USA

**Keywords:** Cell signalling, Molecular biology, Molecular medicine, Oncology

## Abstract

The alkylating agent, temozolomide (TMZ), is the most commonly used chemotherapeutic for the treatment of glioblastoma (GBM). The anti-glioma effect of TMZ involves a complex response that includes G2-M cell cycle arrest and cyclin-dependent kinase 1 (CDK1) activation. While CDK1 phosphorylation is a well-described consequence of TMZ treatment, we find that TMZ also robustly induces CDK1 expression. Analysis of this pathway demonstrates that *CDK1* is regulated by NF-κB via a putative κB-site in its proximal promoter. CDK1 was induced in a manner dependent on mature p50 and the atypical inhibitor κB protein, BCL-3. Treatment with TMZ induced binding of NF-κB to the κB-site as assessed by gel shift analysis and chromatin immunoprecipitation. Examination of a *CDK1* promoter-reporter demonstrated the functional relevance of the κB-site and underlined the requirement of p50 and BCL-3 for activation. Targeted knockdown of *CDK1* or chemical inhibition with the selective CDK1 inhibitor, RO-3306, potentiated the cytotoxic effect of TMZ. These results identify *CDK1* as an NF-κB target gene regulated by p50 and BCL-3 and suggest that targeting CDK1 may be a strategy to improve the efficacy of TMZ against GBM.

## Introduction

Glioblastoma (GBM) is the most common primary malignant brain tumor diagnosed in adults. Standard treatment for GBM involves maximal surgical resection followed by ionizing radiation (IR) and alkylating chemotherapy. Despite aggressive multimodal treatment, overall prognosis for patients with GBM remains poor with an average survival of fourteen to seventeen months^1^. Among chemotherapeutics, the oral alkylator temozolomide (TMZ) has proven to be the optimal choice for adjuvant GBM treatment. Despite its near universal use, many patients experience minimal benefit from the addition of TMZ to their treatment regimen. TMZ primarily mediates its therapeutic effect by forming O^6^-methylguanine (O^6^-MeG) adducts^2^. Following replication, O^6^-MeG lesions preferably mispair with thymine creating a mismatch. This base mismatch activates the mismatch repair (MMR) system that targets the thymine leaving the methylated guanine intact. It has been proposed that successive rounds of such futile repair leads to replication stress and ultimately DNA double strand breaks (DSBs)^3,4^.

CDKs are a family of kinases intimately involved in regulating the cell cycle. The transition between cell cycle stages is tightly controlled by the equilibrium between CDKs, their regulatory subunits, and CDK inhibitors^5^. Among the CDKs, CDK1 regulates the G2-M transition and promotes entry to mitosis by binding cyclin B. Both overexpression and depletion of CDK1 alters coordinated entry into mitosis and can affect the duration of G2-M arrest^6,7^. TMZ promotes phosphorylation and activation of CDK1 thereby modulating cell cycle progression^8,9^. Given the importance of cell cycle regulation to the malignant phenotype, there has been keen interest in targeting CDKs for therapeutic benefit in cancer including GBM^10^.

Nuclear Factor-κB (NF-κB) plays a central role in the cellular response to DNA damage^11^. TMZ modulates NF-κB signaling in a promoter specific manner to both attenuate and augment cell death^12–14^. The mammalian NF-κB transcription factor family is made up of five subunits: p50 (NF-κB1, p105), p52 (NF-κB2, p100), p65 (RelA), cRel, and RelB^15^. NF-κB dimers bind DNA sequences in target genes involved in a wide range of cellular processes. p50, the mature product of *NFKB1,* is found at baseline in the nucleus of most cells^14^. p50 lacks a transactivation domain (TAD) and induces NF-κB-dependent gene expression in association with TAD-containing NF-κB subunits or co-regulators. One of the best described NF-κB co-regulators is B cell leukemia-3 (BCL-3), a protein overexpressed in multiple malignancies^16–18^. BCL-3 is a predominantly nuclear inhibitor κB (IκB) protein that mediates its cellular effects in association with p50- or p52-containing NF-κB dimers^19^.

Although it is known that TMZ promotes CDK1 phosphorylation, on genome-wide analysis we found that *CDK1* was also one of the transcripts most significantly induced by TMZ. In the current report, we examine *CDK1* expression and identify a novel putative κB-site in its proximal promoter. Mechanistic studies demonstrate that in response to TMZ, *CDK1* expression is regulated via the co-operative interaction of p50 and BCL-3. Finally, we find that specific targeting of CDK1 improves the cytotoxic effect of TMZ.

## Results

### TMZ induces CDK1 expression

**Figure 1:**
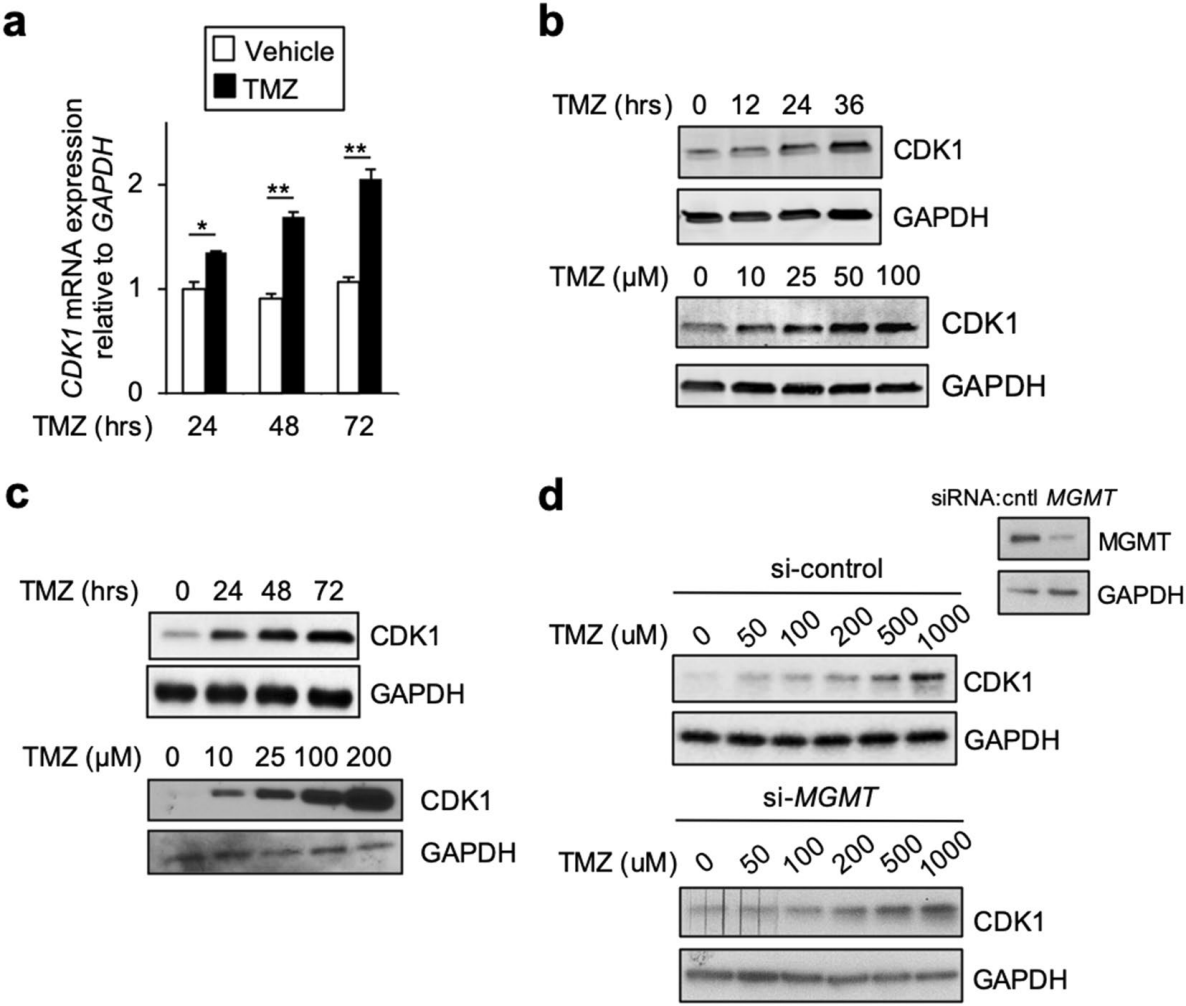
TMZ induces CDK1 expression. (**a**) qPCR analysis of *CDK1* mRNA expression in U87 cells at the indicated times following treatment with vehicle or TMZ (100 μM). Data show mean expression relative to *GAPDH*, ± SD of triplicate samples from three separate biological experiments normalized to vehicle treatment. (**b**–**d**) Immunoblot (IB) with anti-CDK1 or anti-GAPDH antibody. (**b**) U87 cells treated with 100 μM TMZ for indicated time (top) or with the indicated concentration for 24 h (bottom). (**c**) GBM34 cells treated with 100 μM TMZ for indicated time (top) or the indicated concentration for 24 h (bottom). (**d**) T98 cells expressing either si-control or si-MGMT treated with TMZ for 24 h. Inset: immunoblot with anti-MGMT. IB data representative of duplicate experiments. *P < 0.05, **P < 0.01.

We previously reported the genome-wide expression profile of GBM cells following treatment with TMZ (GSE65363)^20^. Analysis of the data revealed that *CDK1* was one of the transcripts most significantly induced by TMZ (adj. *P* value < 0.01). Given the potential importance of CDK1 to the cytotoxic response to TMZ^9^, we further examined *CDK1* expression. Treatment of U87 GBM cells with TMZ induced the expression of *CDK1* mRNA within 24 h (Fig. 1a). Consistent with this, CDK1 protein abundance was also increased by TMZ with a similar time course and in a concentration-dependent manner (Fig. 1b). To examine these findings in a distinct cell line, we incorporated the patient-derived GBM stem-like cell (GSC), GBM34^21^. In GBM34 GSCs, CDK1 was induced by TMZ within 24 h, and this increase was seen at an even lower TMZ concentration than in U87 cells (Fig. 1c). These findings demonstrate that CDK1 expression is up-regulated by TMZ. We next examined whether the MGMT expression regulated the induction of CDK1 by TMZ. To study this, we employed T98 GBM cells that have high MGMT protein, and examined CDK1 following knockdown with SiRNA. Despite excellent MGMT knockdown, no significant difference in the ability of TMZ to induce CDK1 was seen compared to cells expressing a control siRNA (Fig. 1d). This finding suggested that up-regulation of CDK1 by TMZ was not dependent on formation of O^6^-MeG. Given this observation, we also examined CDK1 in response to other DNA damaging agents. CDK1 was upregulated by both etoposide, an agent that induces replication stress, and IR (Supplementary Fig. S1a–c). Together these results indicate that CDK1 is up-regulated by DNA damaging agents.

### TMZ up-regulates CDK1 in a BCL-3 dependent manner

**Figure 2:**
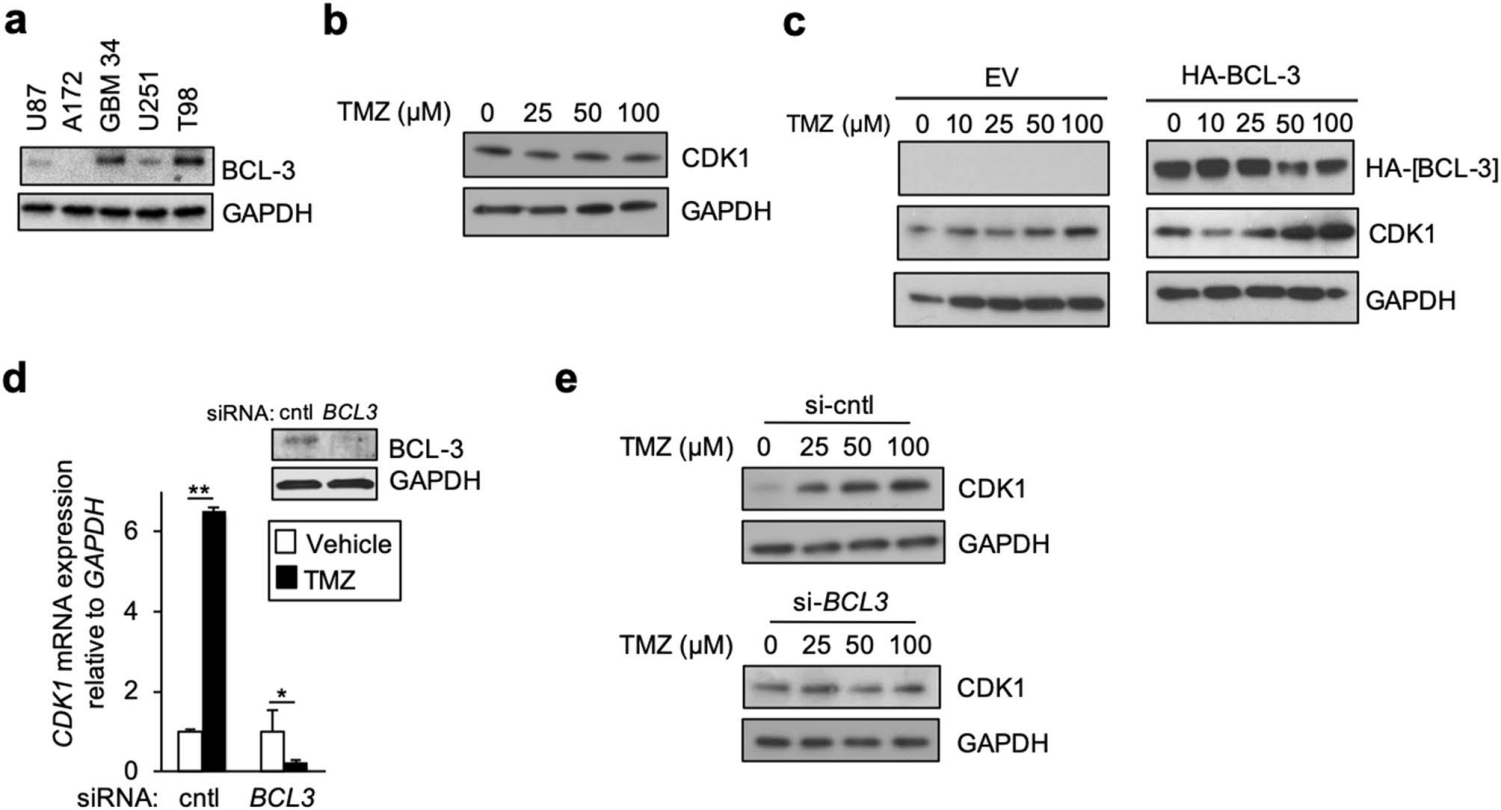
BCL-3 is required for induction of CDK1 by TMZ. (**a**–**c**) IB with anti-CDK1, anti-BCL-3, anti-HA, or anti-GAPDH. (**a**) IB using lysate from the indicated cell line. (**b**) IB in A172 cells following treatment with TMZ (24 h). (**c**) A172 cells expressing either HA-BCL-3 or empty vector (EV) following treatment with the indicated concentration of TMZ (24 h). (**d**) qPCR analysis of mean *CDK1* mRNA in U87 cells transfected with si-control (cntl) or si–*BCL3* treated with TMZ (100 μM, 24 h). Data show mean expression relative to *GAPDH*, ± SD of triplicate samples. Inset: IB with anti-BCL-3. (**e**) IB with anti-CDK1 or anti-GAPDH in U87 cells transfected with si-control or si-*BCL3* following treatment with TMZ for 24 h at indicated concentration. IB data representative of duplicate experiments. *P < 0.05, **P < 0.01.

We previously described a role for BCL-3 in the cytotoxic response to TMZ^16,22^. To examine whether BCL-3 is involved in up-regulation of CDK1 by TMZ, we employed A172 cells that have negligible BCL-3 protein abundance (Fig. 2a). In A172 cells, treatment with TMZ did not increase CDK1 protein (Fig. 2b). On the other hand, expression of exogenous BCL-3 in A172 cells enabled TMZ to up-regulate CDK1 in comparison to cells expressing empty vector (Fig. 2c). This finding was supported by the observation that in U87 cells that have low basal BCL-3, expression of exogenous BCL-3 enables TMZ to induce CDK1 at a substantially lower concentration than in control (Supplementary Fig. S2a). To further examine the requirement of BCL-3, we depleted *BCL3* using siRNA. Knock-down of *BCL3* in U87 cells blocked the increase in *CDK1* mRNA by TMZ (Fig. 2d) and prevented the increase in CDK1 protein (Fig. 2e). This latter finding was recapitulated in GBM34 GSCs where knockdown of *BCL3* attenuated up-regulation of CDK1 by TMZ (Supplementary Fig. S2b). These results indicate that BCL-3 is required for up-regulation of CDK1 by TMZ.

### TMZ up-regulates CDK1 by inducing p50 binding to the *CDK1* promoter

**Figure 3:**
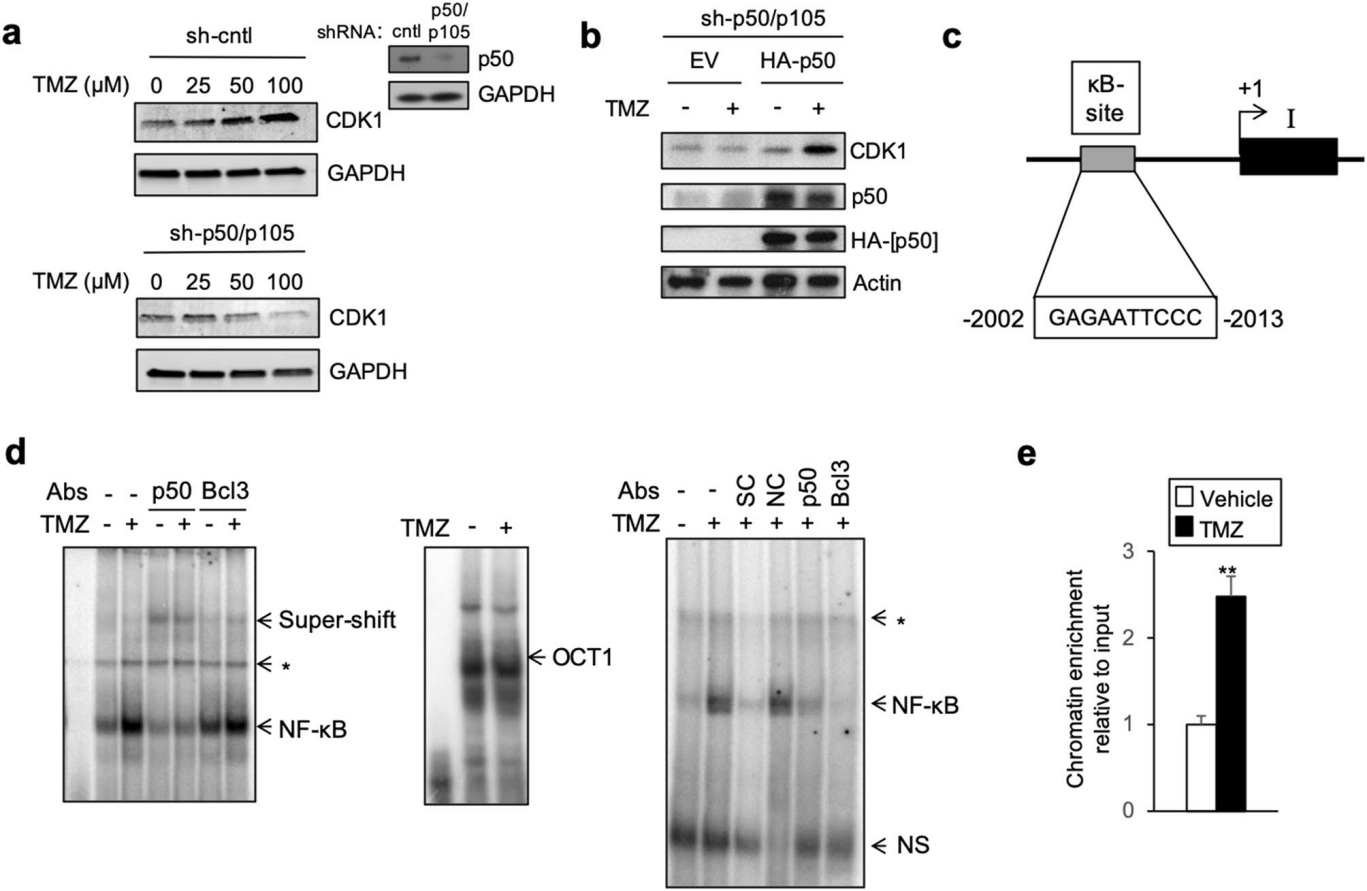
TMZ induces CDK1 expression in a p50-dependent manner and *CDK1* contains a conserved κB-site that binds p50. (**a**,**b**) Immunoblt (IB) with the indicated antibody. (**a**) U87 cells stably expressing sh-control (cntl) or sh-p50/p105 treated with TMZ (24 h). (**b**) U87 cell stably expressing sh-p50/p105 transfected with HA-p50 or empty vector (EV) following treatment with TMZ (100 μM, 24 h). (**c**) Schematic representation of *CDK1* promoter region. (**d**) EMSA with the *CDK1* κB-site probe using nuclear extract from U87 cells treated with TMZ (100 μM, 24 h). Supershift was performed using anti-p50 and anti-BCL-3. NS represents non-specific band. OCT1 EMSA (middle panel) demonstrates equal loading. Competition EMSA (right panel) performed following incubation of κB-site probe with specific and non-specific competitors (SC and NC), respectively. *Represents band not shifted by either anti-p50 or anti-BCL-3. (**e**) qChIP in U87 cells treated with TMZ (100 μM, 24 h). Data represent mean chromatin enrichment using anti-p50 relative to input after controlling for nonspecific binding using anti-histone H1 (positive control) and anti-IgG, normalized to vehicle, ± SD of triplicate samples, repeated. IB data representative of duplicate experiments. **P < 0.01.

BCL-3 primarily mediates its effects in conjunction with p50 or p52-containing dimers^19,23^, and we previously reported that TMZ modulates the interaction of BCL-3 and p50^16^. We therefore examined whether p50 was required for expression of CDK1 by TMZ. For these studies, we used GBM cells stably expressing an sh-RNA targeting the C-terminal of *NFKB1*, the gene encoding p105 the parental protein of p50^13^. Knockdown of *NFKB1* (referred to as sh-p50/p105) in U87 cells blocked the increase in CDK1 by TMZ (Fig. 3a). Notably, targeting a different region of *NFKB1* with a distinct si-RNA also attenuated the increase in CDK1 by TMZ both in U87 cells and patient-derived GBM34 GSCs (Supplementary Fig. S3a,b). Moreover, re-expression of p50 in cells expressing sh-p50/p105 restored the ability of TMZ to induce CDK1 protein (Fig. 3b) indicating that mature p50 itself was sufficient to enable induction of CDK1 by TMZ. These results demonstrate that TMZ up-regulates CDK1 in a p50-dependent manner.

*CDK1* has not previously been reported to be regulated by NF-κB. We searched the promoter and first intron region of *CDK1* (NCBI Gene ID: 983) for potential NF-κB consensus elements. A 10-nucleotide sequence sharing 86% homology with the canonical κB binding site was identified in the proximal promoter (Fig. 3c). To examine binding of NF-κB to this putative κB sequence, nuclear lysate from U87 cells was incubated with an oligonucleotide probe bearing the κB-site. Gel shift assay (EMSA) using the κB probe demonstrated a unique band that was increased following treatment with TMZ (Fig. 3d). As a specificity control, we examined binding to an OCT1 probe and noted no change in the band. Supershift studies demonstrated that both anti-p50 and anti-BCL-3 antibodies shifted the NF-κB band indicating that both factors were present in the protein/DNA complex (Fig. 3d). In addition, analysis of the band using specific and non-specific competitor DNA (SC and NC, respectively) demonstrated loss of the band with SC but not NC indicating that the supershifted band is specific for the κB-site (Fig. 3d, right panel). The above findings suggested that p50 is recruited to the *CDK1* promoter. To examine this in cells, we performed chromatin immunoprecipitation (ChIP) using primers spanning the putative κB-site. p50 was recruited to this region and treatment with TMZ significantly increased enrichment compared to control (Fig. 3e). Notably, no change in recruitment of BCL-3 to this region was seen (Supplementary Fig. S4a). Together, the above findings indicate that p50 binds a putative κB-site in the *CDK1* promoter and that treatment with TMZ increases this interaction. Of note, TMZ did not alter the actual nuclear abundance of p50 or BCL-3 (Supplementary Fig. S4b).

**Figure 4:**
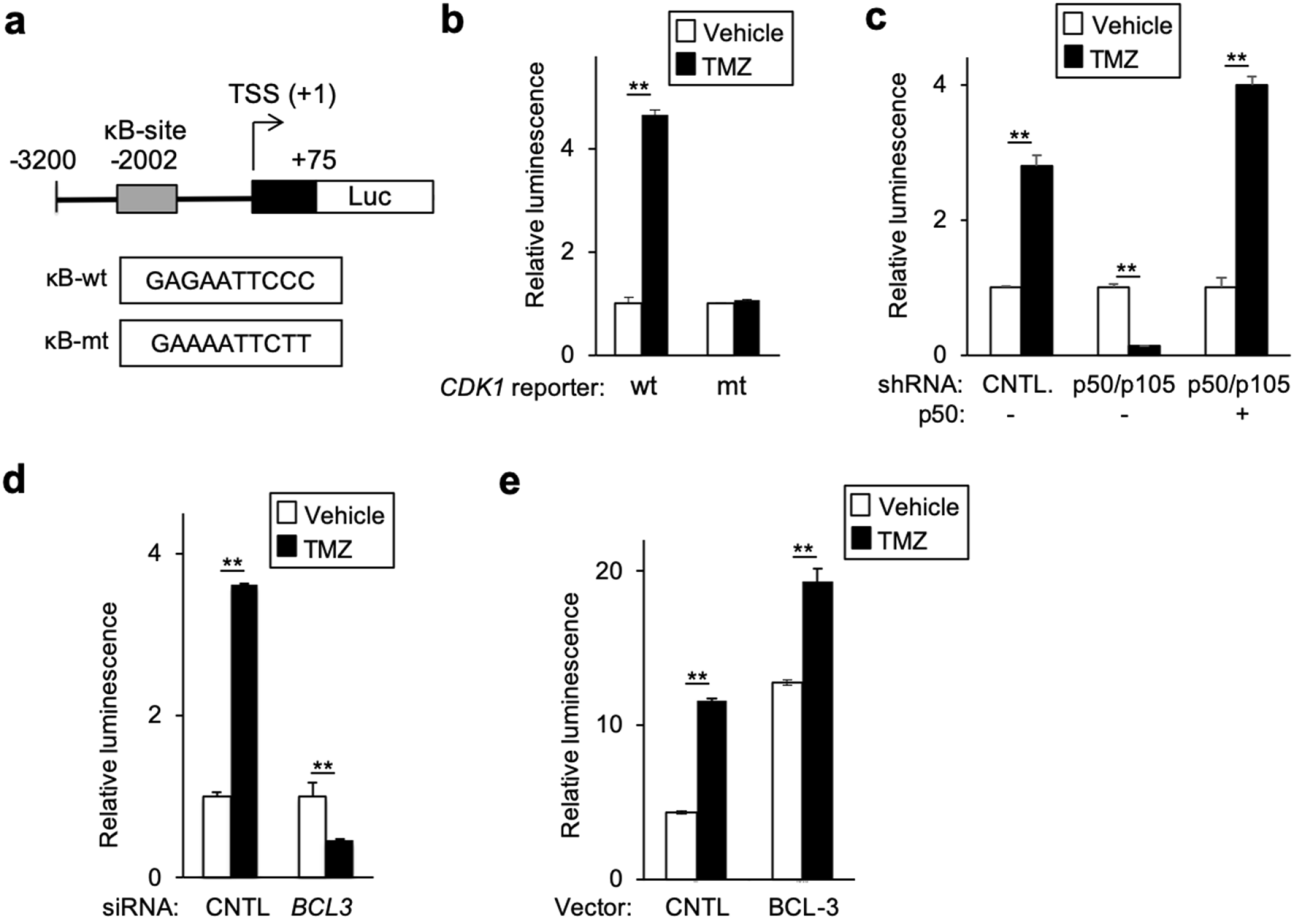
The κB-site is required for activation of a *CDK1* promoter-reporter by TMZ. (**a**) Schematic representation of the 3.2 kbp *CDK1* promoter-reporter. (**b**) Luciferase expression from the indicated reporter relative to *renilla* in U87 cells following treatment with vehicle or TMZ (100 μM, 24 h). (**c**–**e**) Luciferase assays using the wildtype reporter in U87 cells following treatment with TMZ (100 μM, 24 h). (**c**) U87 cells stably expressing sh-control (cntl) or sh-p50/p105, transfected with empty vector (EV) or p50. (**d**) U87 cells transfected with si-control or si-*BCL3*. (**e**) U87 cells transfected with EV or BCL-3. Data represent relative luciferase expression normalized to control, ± SD of triplicate samples, repeated. **P < 0.01.

The binding of p50/BCL-3 to the *CDK1* promoter raised the question of whether this had functional relevance. To examine this, we obtained a luciferase reporter construct containing 3200 nucleotides of the proximal human *CDK1* promoter^24^. The putative κB-site in this reporter was mutated (Fig. 4a) and the response of these constructs to TMZ examined. While TMZ induced luciferase expression from the wildtype reporter, no increase in luciferase was observed from the mutant construct (Fig. 4b). Additionally, while knockdown of *NFKB1* (p50/p105) blocked TMZ-induced expression from the wildtype reporter, re-expression of p50 in cells depleted of p50/p105 enabled TMZ to induce luciferase expression (Fig. 4c). This finding supports the contention that mature p50 is necessary for induction of *CDK1* by TMZ. In addition, consistent with the requirement of BCL-3 for induction of CDK1, knockdown of *BCL3* blocked the increase in luciferase expression following TMZ treatment (Fig. 4d), whereas over-expression of BCL-3 enhanced the activity of the wildtype reporter (Fig. 4e). Together, these results indicate that the putative κB-site binds p50 and BCL-3 and that these factors are necessary for activation of the *CDK1* promoter by TMZ.

### Depletion of CDK1 sensitizes cells to TMZ

**Figure 5:**
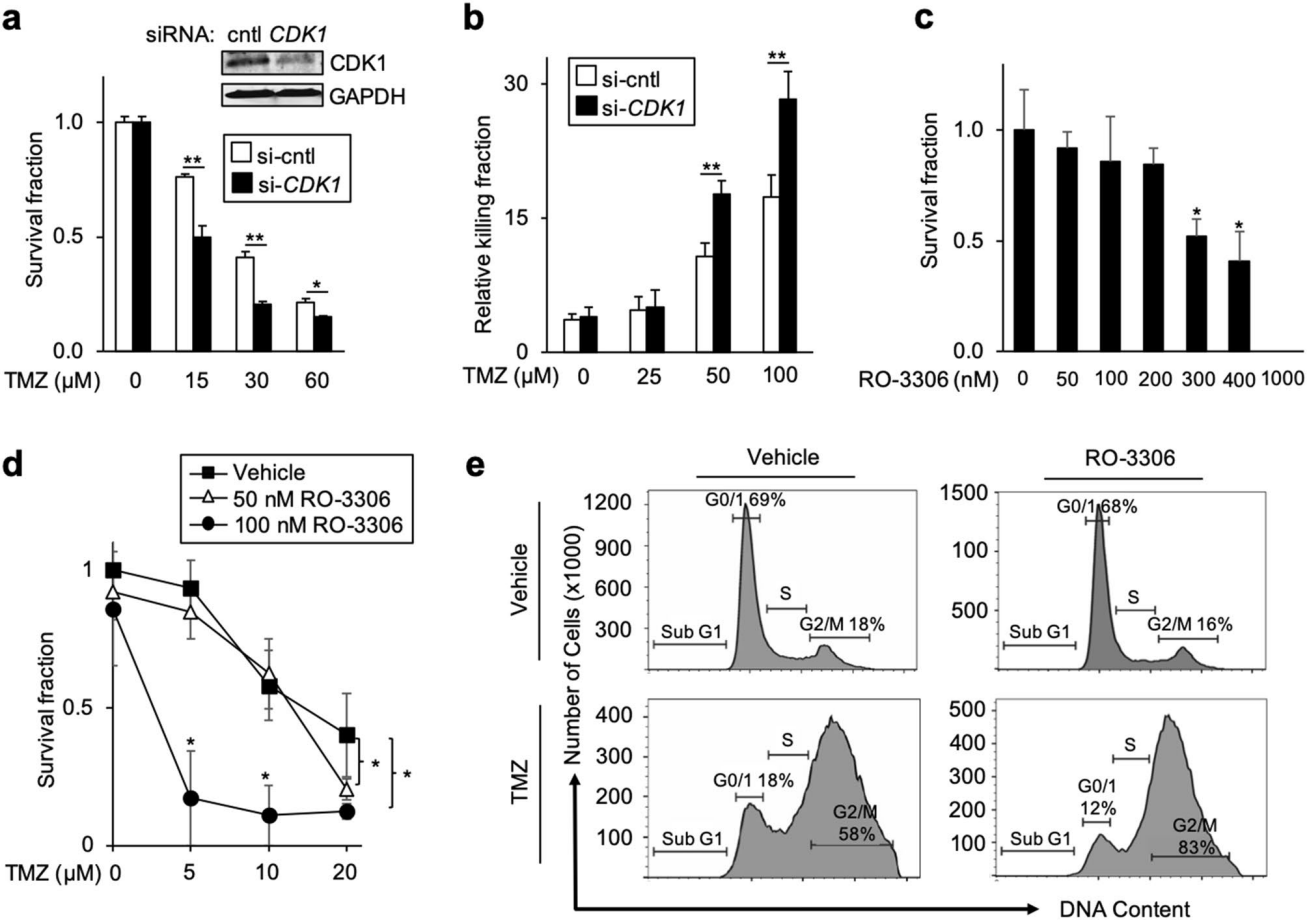
Depletion of CDK1 sensitizes cells to TMZ. (**a**) Clonogenic assay in U87 cells transfected with si-control or si-*CDK1* and treated with TMZ. Inset: Immunoblot with anti-CDK1 and anti-GAPDH. (**b**) Trypan blue assay in GBM34 GSCs transfected with si-control or si-*CDK1* following treatment with TMZ (72 h). (**c**) Clonogenic survival in U87 cells treated with the indicated concentration of RO-3306. (**d**) Clonogenic survival in U87 cells treated with RO-3306 and TMZ. (**e**) Flow cytometric analysis of DNA content after staining with propidium iodide in U87 cells 72 h after treatment with 100 nM RO-3306 and 20 μM TMZ, repeated with similar results. Clonogenic survival data demonstrate mean number of colonies relative to plating efficiency, ± SD of triplicate samples normalized to untreated sample. IB data representative of duplicate experiments. *P < 0.05, **P < 0.01.

Given that CDK1 is induced by TMZ, we examined whether targeting CDK1 modulated the cytotoxic effect of TMZ. Indeed, knockdown of *CDK1* in several established GBM cell lines augmented the anti-glioma effect of TMZ resulting in decreased clonogenic survival following treatment with TMZ (Fig. 5a and Supplementary Fig. S5a,b). Consistent with this, in GBM34 GSCs, depletion of *CDK1* also resulted in increased killing by TMZ (Fig. 5b). Given the role of G2-M arrest to cytotoxicity by TMZ and its regulation by CDK1, we examined the cell cycle under these conditions. While knockdown of *CDK1* alone slightly increased the percentage of cells in G2-M, a finding previously reported in other cell types^25^, loss of CDK1 did not significantly affect the level of G2-M arrest induced by TMZ (Supplementary Fig. S5c).

We next examined whether pharmacologic inhibition of CDK1 also altered the cytotoxic effects of TMZ. While older CDK inhibitors have a broad effect on multiple CDKs^26^, to target CDK1 more specifically, we used RO-3306, a quinolinyl thiaxolinone derivative that inhibits CDK1 activity tenfold more potently than it inhibits CDK2/cyclin E^27^. To identify an appropriate concentration of RO-3306 for use in combination with TMZ, we first examined the cytotoxic effect of RO-3306 alone (Fig. 5c). Notably, while RO-3306 can inhibit CDK1 activity with a K_i_ of 35 nM^27^, we found that at 50 and 100 nM RO-3306 did not induce any significant cytotoxicity of U87 cells (Fig. 5c). We therefore examined clonogenic survival and cell viability using these two concentrations of RO-3306 in combination with TMZ. Both 50 and 100 nM RO-3306 significantly increased the anti-glioma effect of TMZ (Fig. 5d and Supplementary Fig. S5d,e). Notably, the anti-glioma effect of TMZ was more potently enhanced by the higher concentration of RO-3306 (100 nM) than by the lower concentration. In addition, examination of cell cycle revealed that while 100 nM RO-3306 did not significantly affect the cell cycle on its own, it substantially increased TMZ-induced G2-M cell cycle arrest (Fig. 5e).

Finally, in addition to cytotoxicity, cellular invasiveness is also modulated by TMZ and CDK1^28,29^. To examine the effect of combination CDK1 inhibition and TMZ on GBM cell invasion, we used a wound-healing assay. While knockdown of *CDK1* with siRNA or inhibition with RO-3306 alone did not alter the invasiveness of U87 cells, in combination with TMZ, CDK1 inhibition significantly attenuated the invasiveness of these cells (Supplementary Fig. S6a,b). Together, these findings indicate that inhibition of CDK1 sensitizes GBM cells to TMZ and suggest that targeting CDK1 is a potential mechanism to potentiate the anti-glioma effect of TMZ.

## Discussion

Virtually every patient diagnosed with GBM receives the alkylating agent TMZ. In examining the genome-wide expression profile of GBM cells treated with TMZ, we found that *CDK1* is one of the most significantly induced transcripts. While it was previously reported that TMZ promotes phosphorylation of CDK1^8^, a sustained increase in CDK1 protein has not previously been described. We found an increase in CDK1 mRNA and protein within 24 h of TMZ treatment. This increase in CDK1 in response to TMZ was not dependent on cellular MGMT status suggesting that O^6^-MeG lesions are not required for up-regulation of CDK1. While this was unexpected, TMZ induces a variety of DNA lesions^2^ that might contribute to CDK1 up-regulation. We also found that CDK1 protein was induced by other DNA damaging agents. These observations suggest that in the setting of DNA damage, CDK1 is up-regulated either by a number of different lesions or by a common mechanism such as DSBs.

From a mechanistic standpoint, we found that the oncoprotein, BCL-3, is required for induction of CDK1 by TMZ. The requirement of BCL-3 suggested a role for NF-κB in regulating *CDK1* and given that BCL-3 often modulates the downstream response in association with p50-containing NF-κB dimers, we examined the role of p50. Loss of p50 blocked CDK1 expression by TMZ, while re-expression of p50 enabled TMZ to increase CDK1. This latter finding highlighted the importance of mature p50, and not the entire p105 protein, for regulation of CDK1 expression. Interestingly, in numerous assays we found that loss of BCL-3 led to inhibition of CDK1 by TMZ. As p50 lacks a transactivation domain and is often inhibitory, it is possible that in the absence of BCL-3, p50 dimers inhibit *CDK1* promoter activity and mRNA expression. Consistent with regulation by NF-κB, we identified a putative decameric κB-site within the proximal *CDK1* promoter. This sequence was examined using gel shift studies and found to bind a protein complex containing p50 and BCL-3. Treatment with TMZ increased the intensity of this band suggesting that TMZ induced the binding of NF-κB to the κB-site. Support for this was seen with ChIP analysis where we found that TMZ increased p50 recruitment to the region containing the κB-site. Despite the increase in p50 binding, treatment with TMZ did not alter the amount of p50 in the nucleus suggesting that the changes seen on gel shift and ChIP were due to an alteration in p50 DNA binding. This finding is consistent with prior reports indicating that TMZ induces a change in p50 chromatin recruitment^13^. However, unlike prior studies where treatment blocked or didn't alter p50 DNA binding^20,22^, with the *CDK1* promoter, TMZ increased p50 chromatin binding. This variability in p50 chromatin recruitment further supports the observation that post-translational modification of p50 regulates its DNA binding as a function of primary sequence of the κB-site^12^.

The binding of NF-κB to the *CDK1* κB-site raised the question of whether this cis-element was also functional. To examine this, we mutated the κB-site in a *CDK1* promoter-reporter and found that it blocked luciferase expression in response to TMZ. Expression from this reporter was also dependent on both p50 and BCL-3. Together, these findings underline the functional relevance of the κB-site in regulating CDK1 expression. The requirement of NF-κB for CDK1 expression is supported by the recent finding that a novel IκB kinase-β inhibitor down-regulated CDK1 expression and promoted G2-M cell cycle arrest^30^. Also, the requirement of p50 is consistent with a study suggesting that Krueppel-like factor 5 induced expression of CDK1 in association with p50^31^.

The importance of CDK1 to cell proliferation and the propensity of TMZ to induce CDK1 suggested that targeting this kinase might have potential in improving the efficacy of TMZ. We found, as have others^32^, that knockdown of *CDK1* improved the cytotoxicity of TMZ against GBM cells. To examine this strategy using a chemical inhibitor, we chose a newer generation CDK1 inhibitor, RO-3306, a small, bioactive molecule that is cell permeable^27^. We found that at concentrations that inhibit CDK1, RO-3306 had no cytotoxic effect on its own but sensitized several GBM cells to TMZ. While prior studies in GBM have examined general CDK inhibitors such as flavopiridol or roscovitine^33–35^, more specific targeting of CDK1 is uncommon and not previously described with TMZ. Of note, the combination of TMZ and CDK1 inhibition also attenuated the migratory capacity of GBM cells in vitro*.* Unfortunately, it was previously reported that RO-3306 is rapidly cleared from plasma^36^, a finding that limits its ability to cross the blood–brain-barrier (BBB).

In conclusion, we identify *CDK1* as a factor up-regulated by TMZ in GBM cells by a mechanism involving NF-κB signaling. While it is recognized that DNA damage activates CDK1 by phosphorylation, this work adds to the literature on CDK1 demonstrating a mechanism for up-regulation of its protein abundance in response to TMZ. Given the potential efficacy of inhibiting CDK1 as a chemosensitizing strategy, future work developing selective compounds that cross the blood brain barrier might prove effective in improving the management of GBM.

## Methods

### Cell lines, reagents, recombinant proteins and plasmids

Human GBM cell lines U87, A172, T98 and U251 cells were purchased from American Type Culture Collection. The patient-derived GSC, GBM34, was obtained from Dr. Mariano Viapiano (Brigham and Women’s Hospital, Boston, MA) and has been previously described^21^. U87 glioma cells expressing sh-p50/105, targeting the C-terminal of *NFKB1* (to enable re-expression of mature p50), or sh-control were previously described^13^. Cell lines were authenticated by routine morphologic and growth analysis and also by Western blotting. All cell lines were screened for the presence of *Mycoplasma* using the ATCC Universal Mycoplasma Detection Kit (catalog no. 30-1012K) every 4 months. All cells were kept in culture for no longer than 2 months. TMZ and RO-3306 were obtained from Sigma-Aldrich (St. Louis, MO, USA).

### RNA interference

The following siRNA constructs were obtained: si-*CDK1* (s463, Ambion), si-*BCL3* (M-003874-02, Dharmacon), si-*NFKB1* (p50/105) (AM16708, ThermoFisher), and si-control (D-001210-03, GE Dharmacon). All siRNA constructs were transfected at a concentration of 200 nmol/L for 48 h or as noted in the legend using Oligofectamine (Invitrogen, Life Technologies, Grand Island, NY, USA).

### Quantitative real-time polymerase chain reaction (qPCR) and quantitative chromatin immunoprecipitation

Total RNA was isolated and qPCR performed as described^13^. The primers used were: *CDK1* (sense ATGGAAGATTATACCAAA, antisense GGAAAGAAACTCAAA), and *GAPDH* (sense CTTCACCACCATGGAGAAGGC, antisense GGCATGGACTGTGGTCATGAG). Relative expression data is shown as the average of each experiment run in biological triplicate. Non-transcribed controls were included in each run and expression was normalized to 1*GAPDH.*

Quantitative chromatin immunoprecipitation (qChIP) was performed following IP with the indicated antibodies. qPCR was carried out using primers for human *CDK1* that span the region encompassing the putative κB-site (sense GGGTTCTCACATTGCCCTGT, anti-sense TCCAAAGTGCAACACTGTGC). The change in DNA enrichment for each IP condition was determined relative to input DNA. To control for non-specific binding, anti-p50 data was subtracted from anti-H1 results (anti-IgG showed no binding) as previously described^12^.

### Immunoblot and electrophoretic mobility shift assay (EMSA)

Immunoblotting was performed using whole cell lysate as previously described^37^. Primary antibodies used include: anti-p50 (sc7178, Santa Cruz Biotechnology), anti-GAPDH (sc-137179,), anti-CDK1 (sc-54), anti-HA (sc-7392), anti-actin (sc-1616). Unedited, full-length immunoblots are provided in Supplementary Information (Supplementary Figs. S7–Fig. S15). Several immunoblots were cut prior to antibody hybridization for reagent conservation. To identify the κB-site, the program TFSEARCH was used and a sequence with 86% homology with the canonical κB binding site was identified. For EMSA, nuclear fraction was isolated, a double-stranded oligonucleotide (5′-CGCTGAAGAGAATTCCCAAGGC-3′) (IDT) containing the decameric κB-consensus sequence was end labeled with [ɣ-^32^P] ATP and used as a probe, and the assay performed as previously described^13^. Supershift assays were performed using antibodies specific to p50 or BCL-3. Competition was performed by pre-incubating the mixture with cold specific and non-specific DNA probe.

### Luciferase assay

A 3200 bp region of the *CDK1* proximal promoter and coding region was previously cloned upstream of the firefly luciferase DNA in the expression vector Δ5′ PSV 2^24^. This vector was a kind gift from Christopher Glass (University of Southern California) and contains the putative κB-site. This κB-site was mutated using the QuikChange Lightning Site Directed Mutagenesis Kit (Agilent, Santa Clara, CA, USA) (GAGAATTCCC to GAAAATTCTT). Cells were co-transfected with the indicated reporter construct and *Renilla reniformis* and relative luciferase activity measured after treatment using the Dual-Luciferase Reporter Assay kit (Promega) as previously described^37^. All experiments were performed in triplicate and repeated.

### Clonogenic survival assay

Cell were untransfected or transfected with indicated si-RNA for 48 h. Cells were counted, plated and allowed to attach overnight. 12 h after plating, cells were treated with TMZ. For assays with RO-3306, cells were treated with RO-3306 for 12 h, and then treated with TMZ. After 10 days, colonies were fixed with glutaraldehyde (6.0% v/v), stained with crystal violet (0.5% w/v) and counted using a stereomicroscope^38^. The surviving fraction was then calculated based on the plating efficiency of untreated cells.

### Trypan blue killing assay

Where indicated, cells were initially transfected with si-RNA for 48 h. Cells were counted and plated and allowed to attach overnight. 12 h after plating, cells were treated with TMZ. For assays with RO-3306, cells were treated with RO-3306 for 12 h, and then treated with TMZ. Cells were harvested after 3 or 5 days as indicated and stained with trypan blue (0.4%). Living and dead cells were then counted to determine the killing fraction.

### Flow cytometry analysis of fractional DNA content

U87 cells were plated overnight and treated with 20 μM TMZ and 100 nm RO-3306. As previously described^39^, at 72 h, the cells were washed in PBS and fixed in ice-cold 70% (v/v) ethanol. The cells were washed twice, incubated in RNase (1 mg/mL) for 30 min, and then incubated in propidium iodide solution (100 μg/mL) for 30 min. Flow cytometric analysis was performed on a FACSort instrument (Becton Dicksinson Immunocytometry Systems, San Jose, CA), and the data were analyzed using the CellQuest software (Becton Dickinson). Experiment was performed in duplicate.

### Scratch wound assay

Cell invasion was determined using a scratch-wound assay. Cells were transfected with the indicated siRNA for 48 h and then transferred to six-well plates. After 48 h, cell density approached 80%. Cells were subsequently cultured in serum-free media for 24 h. Using a 200 μl sterile pipette tip, parallel lines were marked in the cell monolayer to form wound gaps. Cells and wound width were captured by photograph at 0 and 36 h. The relative area of the scratch was analyzed using ImageJ to determine the wound migration distance.

### Statistical analysis

Data are expressed as a mean ± SD and significance determined as *P* < 0.05 using a two-tailed Student *t* test.

## Supplementary Information

Supplementary Information.

## Data Availability

All data generated and analyzed during this study are included in this published article and its Supplementary Information files or are available from the corresponding author on reasonable request.
