# Supplementary material for: CDK1 is up-regulated by temozolomide in an NF-κB dependent manner in glioblastoma
Source: Sci Rep. 2021 Mar 11;11:5665. doi: 10.1038/s41598-021-84912-4 (PMC7952566; doi:10.1038/s41598-021-84912-4)
Supplement: Supplementary file 1 — Supplementary Information. [file 41598_2021_84912_MOESM1_ESM.pdf]

# CDK1 is up-regulated by temozolomide in an NF-κB dependent manner in glioblastoma

**David J. Voce[1,2], Giovanna M. Bernal[1], Kirk E. Cahill[1], Longtao Wu[1], Nassir Mansour[1], Clayton D. Crawley[1], Paige-Ashley S. Campbell[1], Ainhoa Arina[3], Ralph R. Weichselbaum[3] & Bakhtiar Yamini[1,*]**

[1]Department of Surgery, Section of Neurosurgery, The University of Chicago, Chicago, IL 60637, USA.
[2]Department of Neurosurgery, Vanderbilt University Medical Center, Nashville, TN 37232, USA.
[3]Department of Radiation and Cellular Oncology and The Ludwig Center for Metastasis Research, The University of Chicago, Chicago, IL 60637, USA.
*Corresponding author
Email address: byamini@surgery.bsd.uchicago.edu

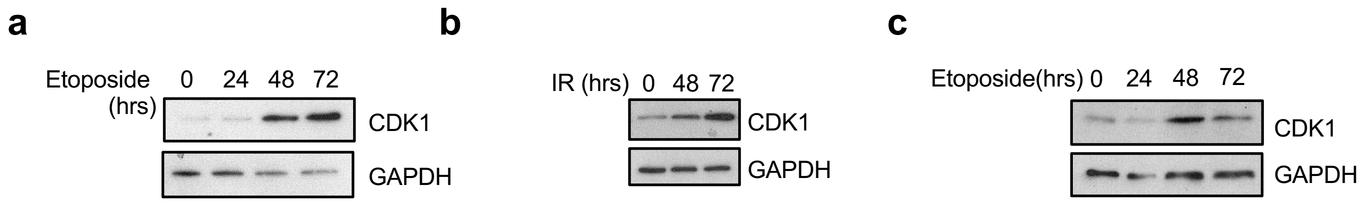


**Supplementary Figure 1.** IR and etoposide induce CDK1 expression. (a-c) Immunoblots (IB) with anti-CDK1 or anti-GAPDH. (a) U87 cells treated with 10 μM etoposide. (b) U87 cells treated to 4 Gy IR. (c) GBM34 cells treated with 10 μM etoposide. IB data representative of duplicate experiments.

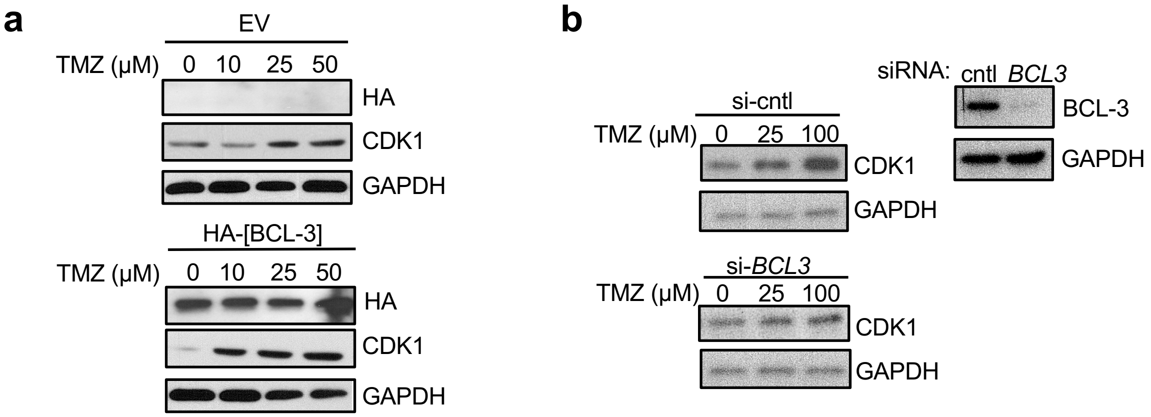


**Supplementary Figure 2.** BCL-3 is required for induction of CDK1 by TMZ.(a-b) IB with anti-CDK1, anti-HA, anti-BCL3, or anti-GAPDH. (a) U87 cells expressing HA-BCL-3 or empty vector (EV) following treatment with TMZ (24 hours). (b) GBM34 cells treated with 48 hours of si-*BCL3* followed by TMZ for 24 hours. IB data representative of duplicate experiments.

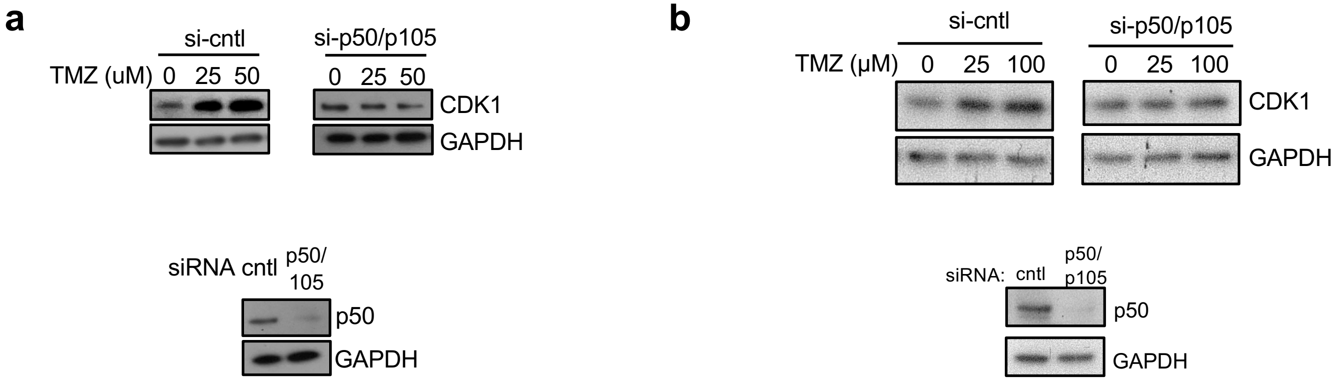


**Supplementary Figure 3.** TMZ induces CDK1 expression in a p50 dependent manner. IB with anti-CDK1 or anti-GAPDH antibody 24 hours following TMZ treatment in (a) U87 cells and (b) GBM34 cells transfected with siRNA-control or si-p50/p105. Lower panels: IB with anti-CDK1 or anti-p50 in cells expressing the indicated si-RNA. IB data representative of duplicate experiments.

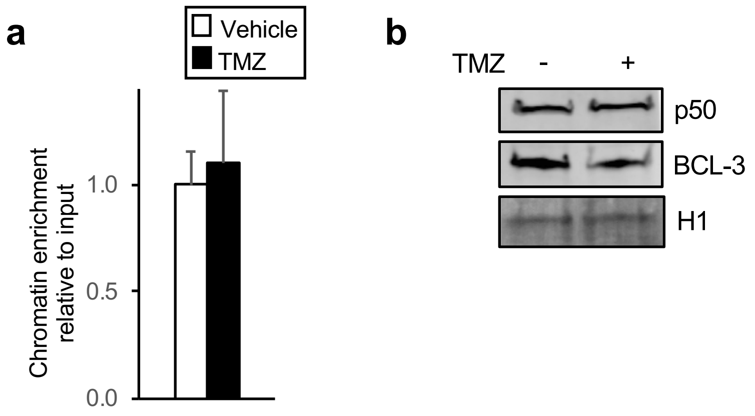


**Supplementary Figure 4.** (a) qChIP in U87 cells treated with TMZ (100 μM, 24 hours). Data represent mean chromatin enrichment with anti-BCL-3 relative to input after controlling for nonspecific binding using anti-histone H1 (positive control) and anti-IgG, normalized to vehicle ±SD of triplicate samples repeated. (b) IB with anti-p50, anti-BCL-3, and anti-H1 in nuclear extract of U87 cells treated with TMZ (100 μM, 24 hours). IB data representative of duplicate experiments.

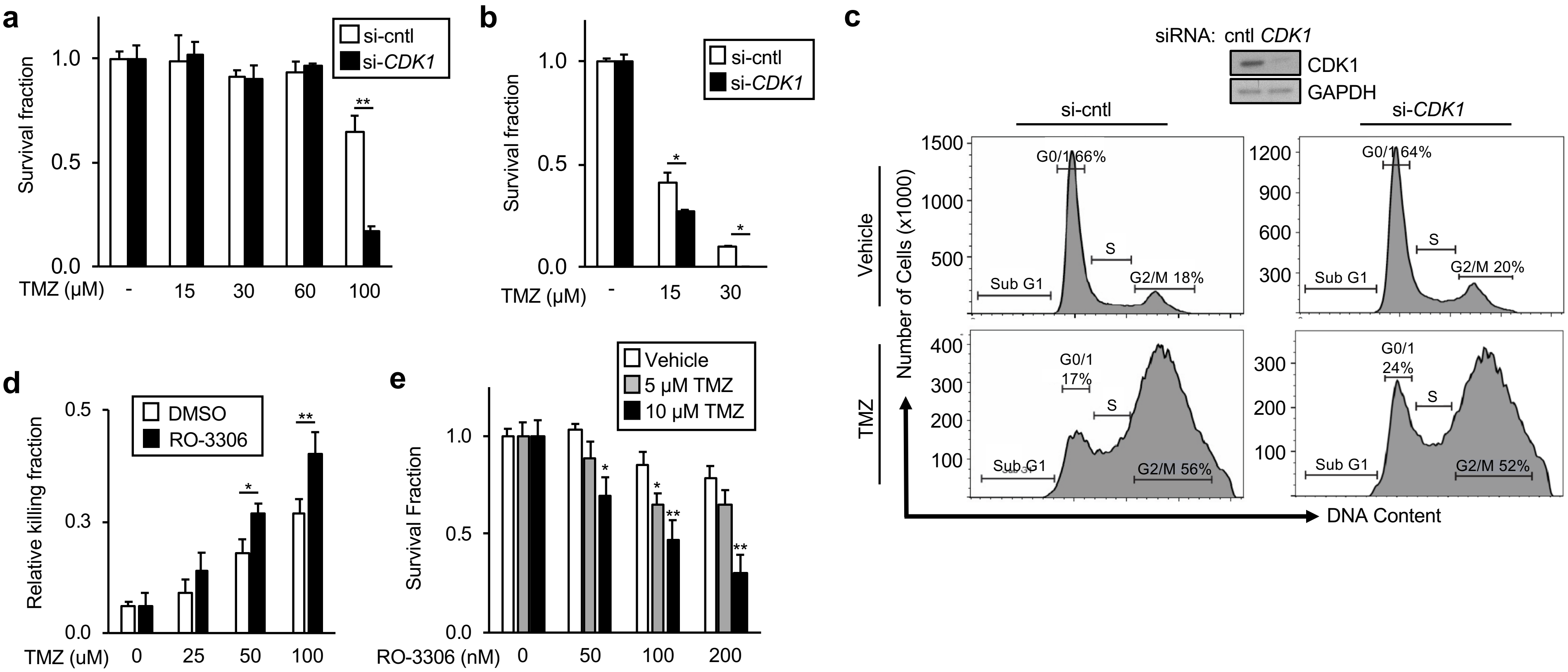


**Supplementary Figure 5.** Targeting CDK1 potentiates TMZ cytotoxicity**.** Clonogenic assay in (a) T98 cells or (b) U251 cells transfected with si-control or si-*CDK1* and treated with indicated concentrations of TMZ. (c) Flow cytometric analysis of DNA fragmentation after staining with propidium iodide 72 hours after treatment with 20 μM TMZ in U87 cells with si-*CDK1*. Top Inset: Immunoblot with anti-CDK1 and anti-GAPDH. (d) Trypan blue killing assay in GBM34 cells and (e) clonogenic assay in U251 cells treated with combination RO-3306 and TMZ. Clonogenic survival data demonstrate mean number of colonies relative to plating efficiency +SD of triplicate samples normalized to untreated sample. IB data representative of duplicate experiments. *, $P < 0.05$, **, $P < 0.01$.

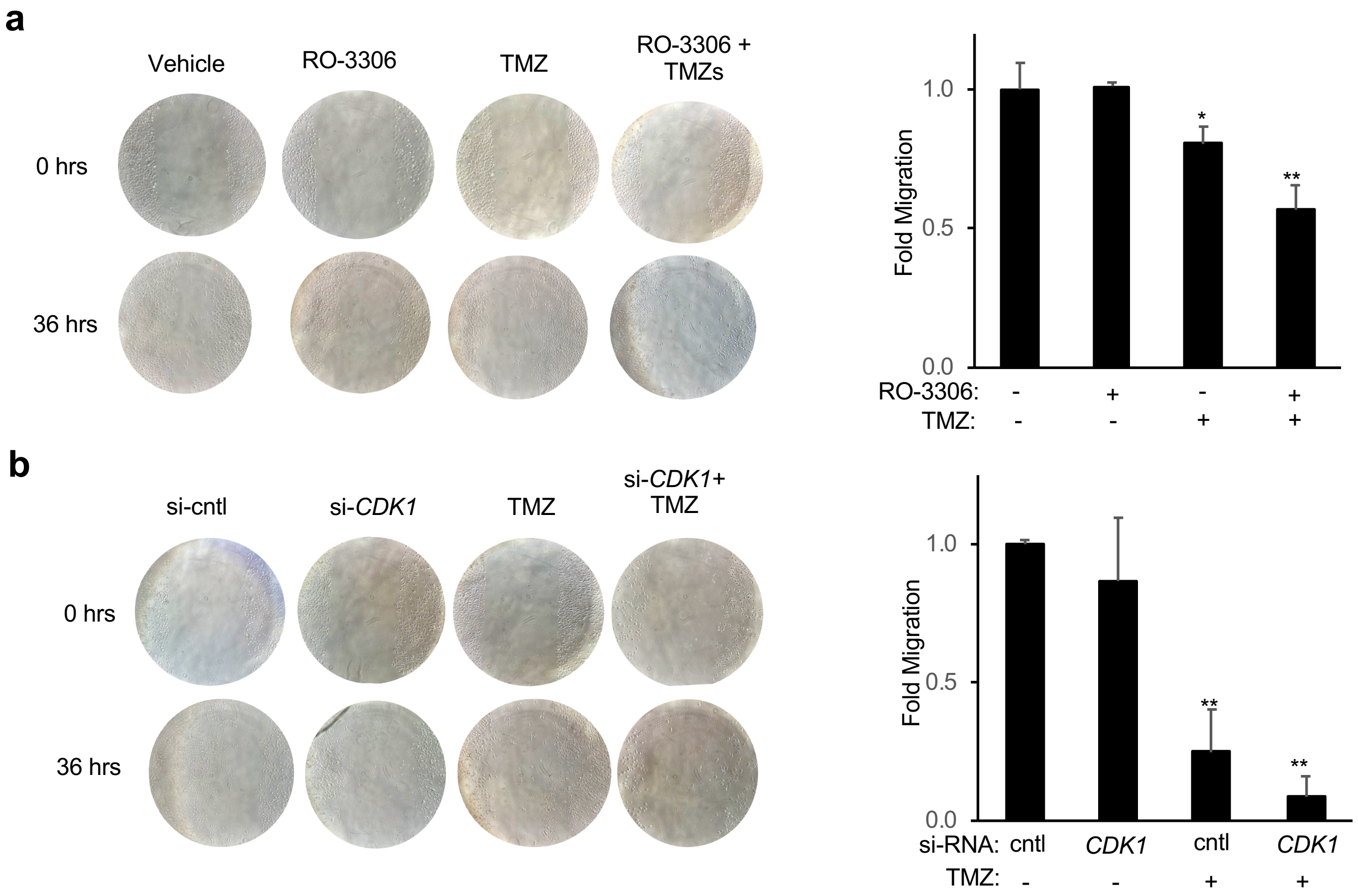


**Supplementary Figure 6.** Combined TMZ treatment and CDK1 inhibition decreases cell migration rate. Cell migration was analyzed in U251 cells after treatment with (a) 100 nM RO-3306 and 100 μM TMZ and (b) si*CDK1* and 100 μM TMZ. Data are presented as mean +̲ SD of three triplicate experiments. *, $P < 0.05$, **, $P < 0.01$.

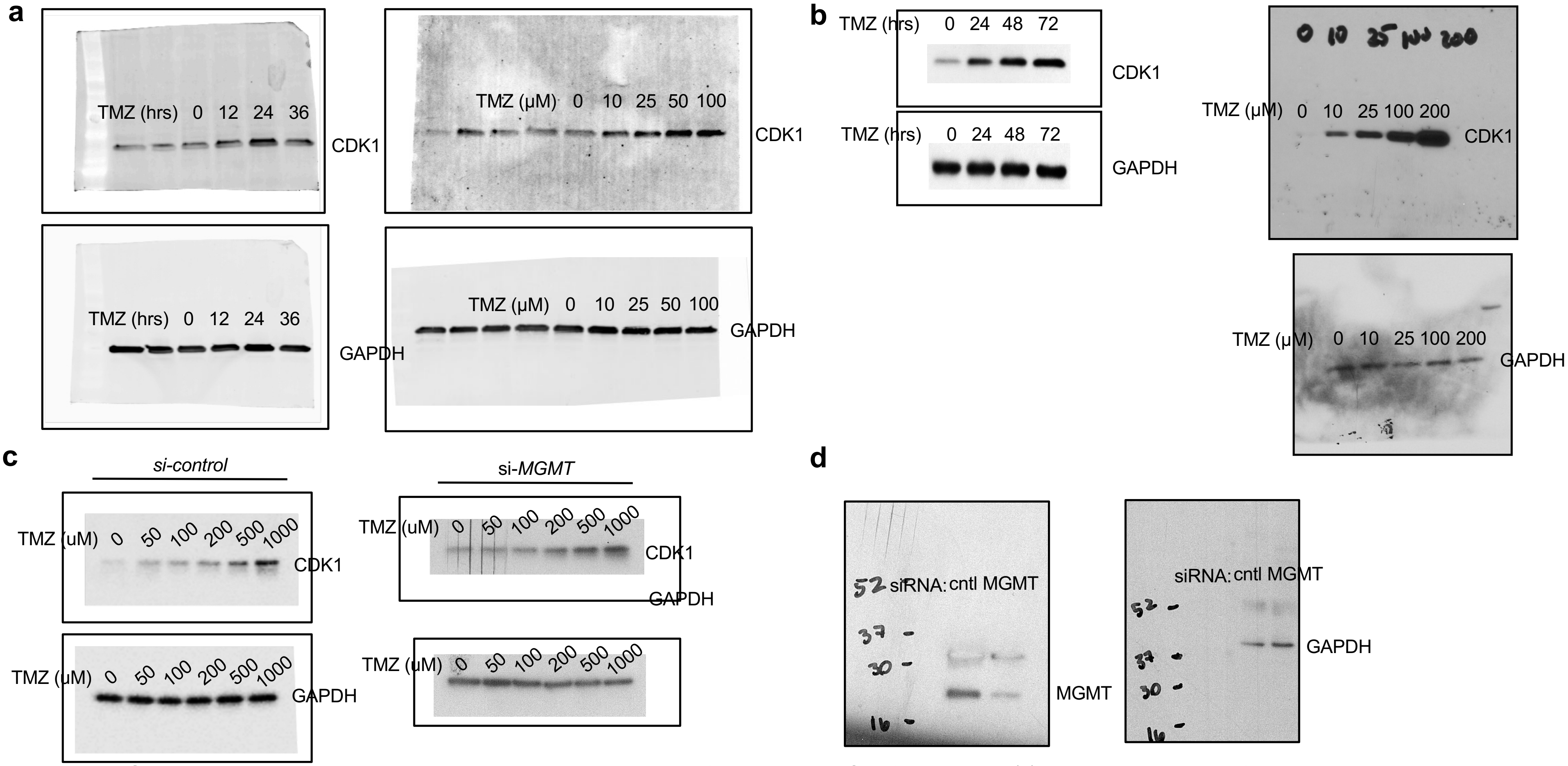


**Supplementary Figure 7.** Expanded blots to accompany Figure 1. TMZ induces CDK1 expression. (a) U87 cells treated with 100 µM TMZ for indicated time (left panels) or with the indicated concentration for 24 hours (right panels). Left panel full length blots were not retained as blots were saved in their cropped version digitally. (b) GBM34 cells treated with 100 µM TMZ for indicated time (left panels) or the indicated concentration for 24 hours (right panels). (c) T98 cells expressing either si-control (left panels) or si-MGMT (right panels) treated with TMZ for 24 hours. Blots cut prior to hybridization. (d) Confirmation of MGMT knockdown in T98 cells expressing si-control or si-MGMT.

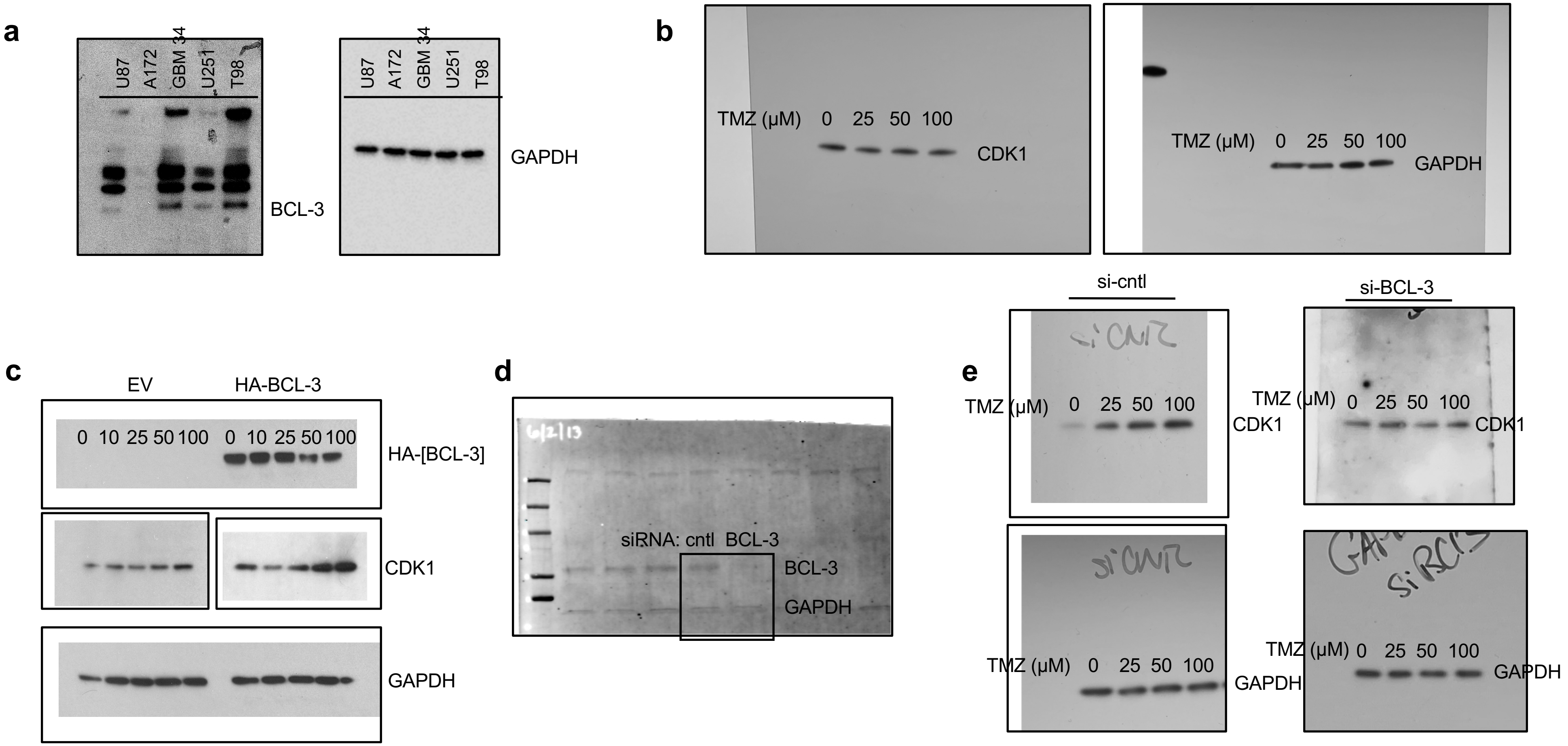


**Supplementary Figure 8.** Expanded blots to accompany Figure 2. BCL-3 is required for induction of CDK1 by TMZ. (a) IB using lysate from the indicated cell line. (b) IB in A172 cells following treatment with TMZ (24 hours). (c) IB in A172 cells expressing either HA-BCL-3 or empty vector (EV) following treatment with the indicated concentration of TMZ (24 hours). Blots were cut prior to hybridization. (d) IB in U87 cells transfected with si-control or si-*BCL3.* (e) IB in U87 cells transfected with si-control or si-*BCL3* following treatment with TMZ for 24 hours at indicated concentration.

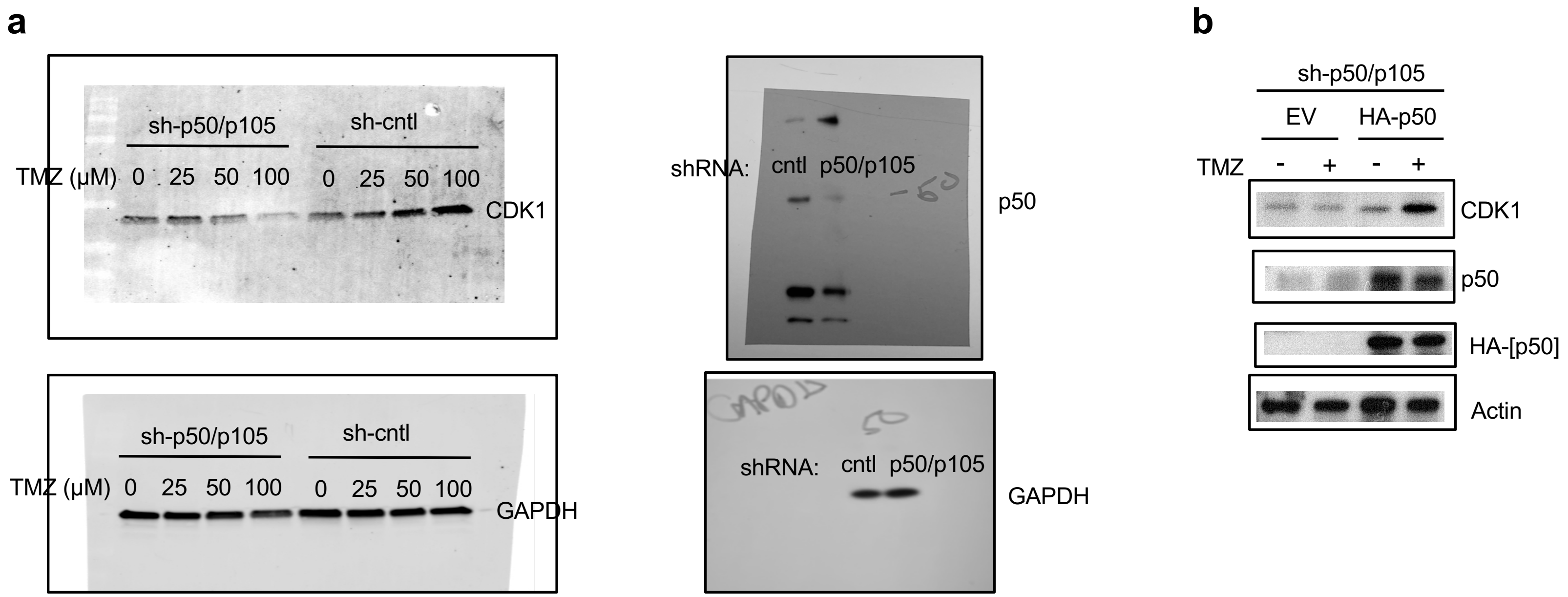


**Supplementary Figure 9.** Expanded blots to accompany Figure 3. TMZ induces CDK1 expression in a p50-dependent manner. (a) U87 cells stably expressing sh-control (cntl) or sh-p50/p105 treated with TMZ (24 hours). (b) U87 cell stably expressing sh-p50/p105 transfected with HA-p50 or empty vector (EV) following treatment with TMZ (100 μM, 24 hrs). Full length blots were not retained as blots were saved in their cropped version digitally.

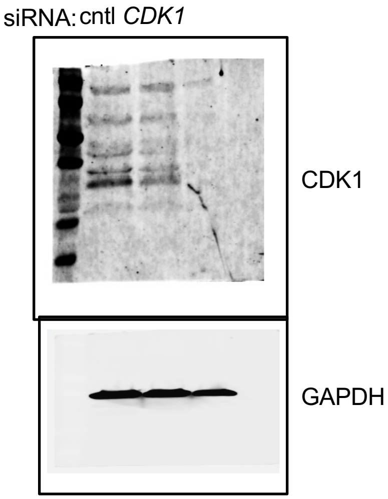


**Supplementary Figure 10.** Expanded blots to accompany Figure 5. U87 cells transfected with si-control or si-*CDK1*

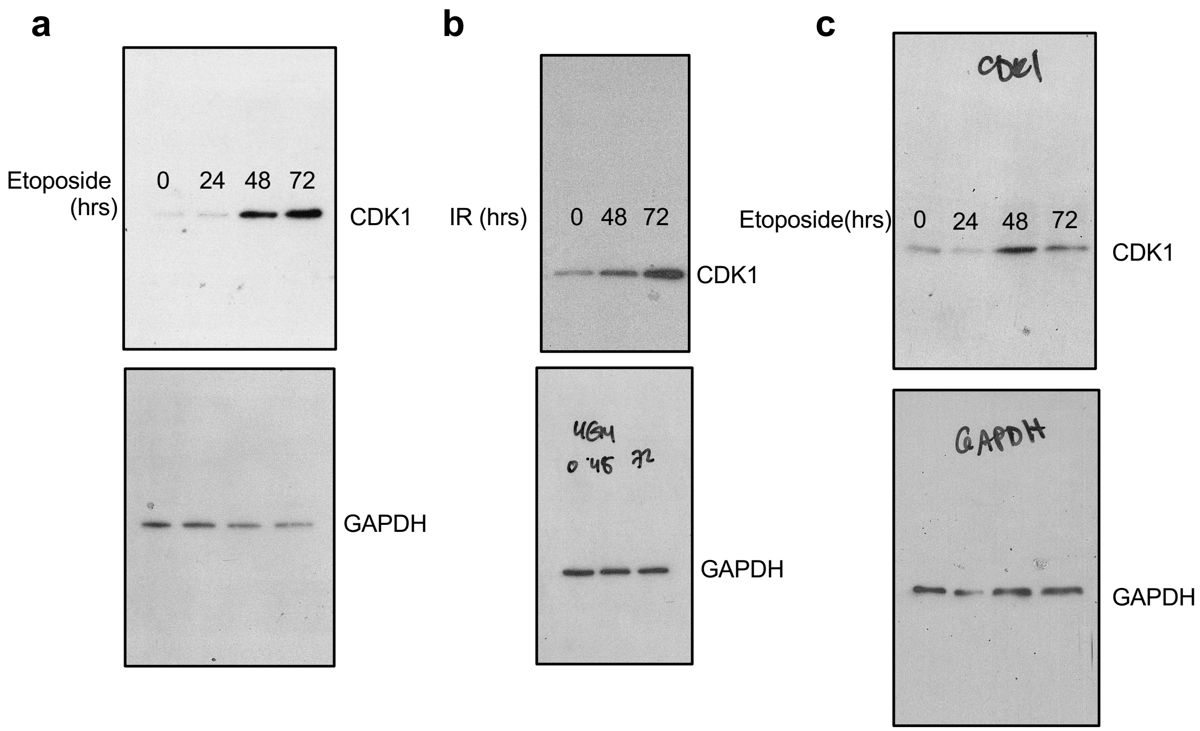


**Supplementary Figure 11.** Expanded blots to accompany Supplementary Figure 1. IR and etoposide induce CDK1 expression. (a) U87 cells treated with 10 μM etoposide. (b) U87 cells treated to 4 Gy IR. (c) GBM34 cells treated with 10 μM etoposide.

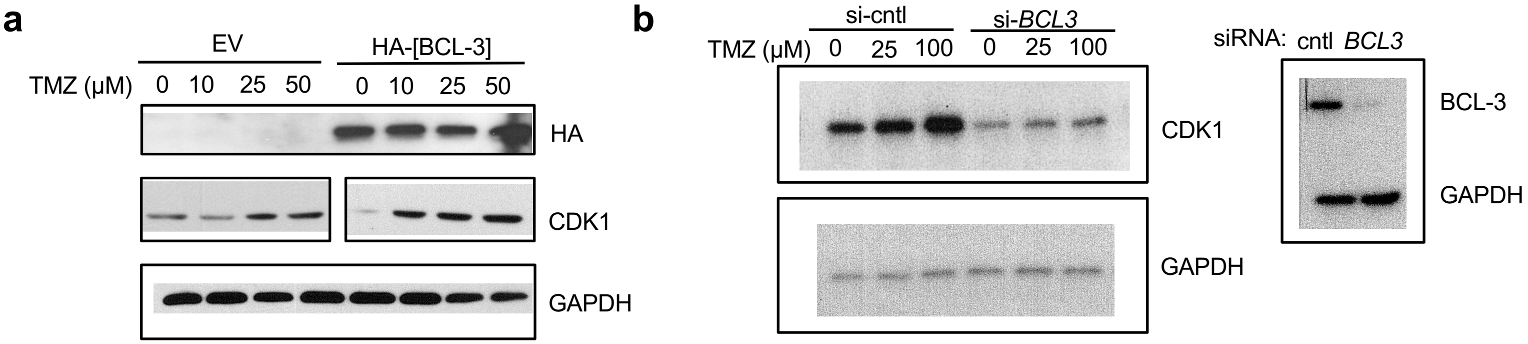


**Supplementary Figure 12.** Expanded blots to accompany Supplementary Figure 2. BCL-3 is required for induction of CDK1 by TMZ. (a) U87 cells expressing HA-BCL-3 or empty vector (EV) following treatment with TMZ (24 hours). Full length blots were not retained as blots were saved in their cropped version digitally. (b) GBM34 cells treated with 48 hours of si-*BCL3* followed by TMZ for 24 hours. Blots were cut prior to hybridization.

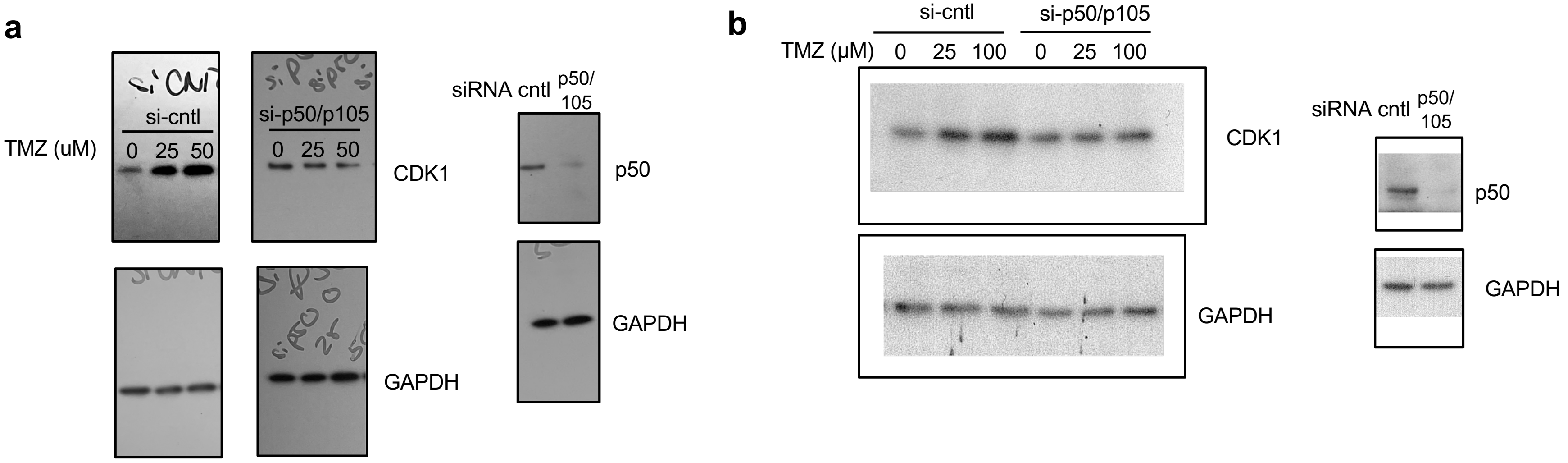


**Supplementary Figure 13.** Expanded blots to accompany Supplementary Figure 3. TMZ induces CDK1 expression in a p50 dependent manner. (a) U87 cells and (b) GBM34 cells transfected with siRNA-control or si-p50/p105 and treated with TMZ. Blots were cut prior to hybridization.

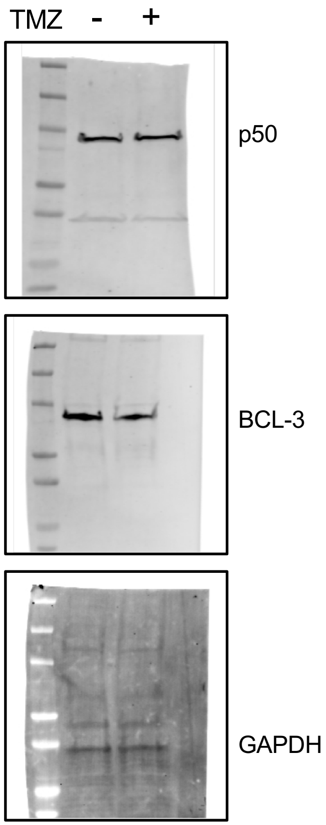


**Supplementary Figure 14.** Full length blot to accompany Supplementary Figure 4. IB with anti-p50, anti-BCL-3, and anti-H1 in nuclear extract of U87 cells treated with TMZ (100 μM, 24 hours).

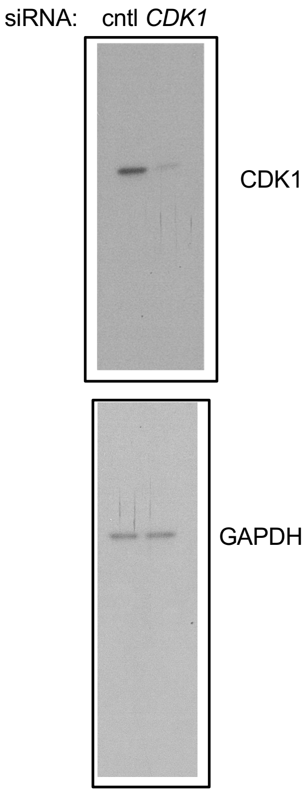


**Supplementary Figure 15.** Expanded blots to accompany Supplementary Figure 5. Immunoblot with confirmation of CDK1 knockdown following siRNA treatment.
